# Unveiling Superior Fracture Toughness in MnCoSb Half-Heusler Alloy: A First-Principles Guide for Designing Damage-Tolerant Functional Materials

**DOI:** 10.3390/molecules31121994

**Published:** 2026-06-07

**Authors:** Ai Qin, Shao-Bo Chen, Lin-Zi Tu, Jia-Hao Wang, Wan-Jun Yan, Tinghong Gao, Kuang-Min Gao, Jing Zhao

**Affiliations:** 1 College of Electronic and Information Engineering, Anshun Uniνersity, Anshun 561000, China; aiqin2025@yeah.net (A.Q.); 13765306886@163.com (L.-Z.T.); jiahaowang2000@yeah.net (J.-H.W.); b20200713@yeah.net (K.-M.G.); zhaojing9421@asu.edu.cn (J.Z.); 2 School of Physics and Electronic Science, Zunyi Normal University, Zunyi 563000, China; yanwanjun7817@163.com; 3 Institute of Advanced Optoelectronic Materials and Technology, College of Big Data and Information Engineering, Guizhou University, Guiyang 550025, China; gaotinghong@sina.com

**Keywords:** mechanical properties, fracture toughness, first-principles calculations, Half-Heusler alloys

## Abstract

In this study, the stability, electronic, structural, and fracture toughness, and mechanical properties of the Half-Heusler(HH) alloys MnCoSb, MnCoAs, MnCoP, and MnNiSb were comprehensively investigated using first-principles calculations based on density functional theory (DFT). The calculated results reveal that all four alloys exhibit half-metallic characteristics, characterized by the presence of a substantial band gap in the spin-down channel. The phonon spectra and negative formation energies confirm that these alloys possess both dynamic and thermodynamic stability. The Born criteria further validate the structural stability in terms of mechanical properties. Three-dimensional representations of the Young’s modulus, bulk modulus, and shear modulus for the four alloys indicate that MnCoP exhibits the most pronounced anisotropy. The overall fracture toughness of the alloys ranges from 1.58 MPa·m^1/2^ to 2.63 MPa·m^1/2^, which falls within the typical range for half-metallic materials, albeit at the lower end, attributable to the relatively ductile nature of the four alloys. Although the two methods yield different absolute values, the explicit crack model (Method I) is considered more reliable for anisotropic systems because it directly simulates crack propagation and accounts for local relaxations, while the empirical formula (Method II) provides a useful reference for high-throughput screening. Among the alloys, MnCoSb demonstrates a superior mechanical performance, with *K*_IC_ values of 2.63 MPa·m^1/2^ and 1.58 MPa·m^1/2^ and brittleness indices *M* of 8.97 and 14.94, indicating excellent damage tolerance compared to the other three alloys. In contrast, MnCoP exhibits higher brittleness and lower mechanical reliability, with *K*_IC_ values of 2.00 MPa·m^1/2^ and 1.63 MPa·m^1/2^ and higher *M* values of 13.83 and 16.99. This study provides quantitative predictions of fracture toughness and establishes a relationship between microscopic and mechanical properties. These findings offer a theoretical foundation for the application of damage-tolerant HH alloys in fields such as spintronics and magnetism.

## 1. Introduction

Materials are increasingly integral to daily life, with diverse applications in aerospace, high-end energy manufacturing equipment, shipbuilding, automotive engineering, and transportation infrastructure. The demand for advanced materials is gradually shifting toward multifunctional integration. Heusler alloys have attracted the attention of the scientific community for decades [1] due to their tailorable physicochemical properties, making them a subject of extensive research. Their unique characteristics render them highly suitable for advanced applications in spintronics, thermoelectrics, and optoelectronics [2,3,4,5,6]. Early research on Heusler alloys has been summarized by F. Dahmane et al. [7]. A foundational discovery by German mining engineer Friedrich Heusler showed that specific combinations of non-ferromagnetic elements, such as Mn and Cu with Sn, Al, or Sb, could exhibit ferromagnetic behavior [8]. This phenomenon has since spurred extensive research on Heusler alloys, leading to the discovery of a range of remarkable properties [9], including ferromagnetism, significant spin-polarization effects [10], and promising semiconducting thermoelectric characteristics [11,12]. Structurally, HH compounds, which combine excellent functional properties (e.g., thermoelectric performance) with good high-temperature strength, are currently a research focus. Among the thousands of known HH compounds, this class of materials exhibits both superior thermoelectric conversion efficiency and outstanding mechanical reliability, with particular attention paid to their fracture resistance under coupled high-temperature and stress conditions. As a new generation of functional materials, HH compounds have gained growing attention due to their unique magnetic and electronic properties, as well as excellent performance in terms of structural stability, mechanical strength, and thermal transport. Furthermore, their half-metallic character endows them with great potential for cutting-edge fields like spintronics [13,14], making them a key focus of both fundamental and applied research.

The structural integrity and service life of HH alloys in practical applications are critically dependent on mechanical properties such as fracture toughness and hardness. A significant advantage of these materials is their capacity to tailor mechanical performance through rational design. Chemical substitution and structural tuning, two strategic modification approaches, constitute a fundamental method for the intentional regulation of material property profiles, enabling the targeted adjustment of material characteristics by altering their basic composition and core structure, thus facilitating their incorporation into durable devices requiring high mechanical stability [15]. Additionally, HH compounds have emerged as promising, cost-effective, and eco-friendly materials for thermoelectric (TE) applications. They are widely used in power generators for waste heat recovery systems and cooling devices utilizing the Peltier effect, which realizes energy conversion by exploiting temperature gradients [16,17]. The inherent tunability and multifunctionality of HH compounds have become the main driving force for the ongoing exploration of novel HH systems, with the optimization of TE properties as a central research objective [17]. Beyond superior functional properties, excellent mechanical characteristics, especially fracture toughness, are essential to ensure the long service life of advanced materials. However, current research on Heusler alloys has mainly focused on their excellent thermoelectric and optical properties, with relatively limited investigations into their fracture toughness. This research gap hinders the accurate prediction of the damage tolerance of such materials in practical service. Along with the continuous development of novel alloy systems, considerable research efforts are also devoted to improving the mechanical properties of mature thermoelectric (TE) materials, as their practical application and commercial scalability require viable candidates to meet key prerequisites, including facile synthesis, high structural stability, and tunable electronic and phonon properties [17]. Although previous studies have extensively investigated the thermoelectric and optical properties of HH compounds [18,19,20], research on their fracture toughness, a critical parameter determining the reliability of structural applications, remains notably scarce. To the best of our knowledge, only a small number of studies have reported the evaluation of fracture toughness for HH alloys.

In light of the above considerations, the present study conducts a comprehensive theoretical investigation on the critical mechanical characteristicsparticularly fracture toughnessof HH compounds MnNiSb and MnCoZ (Z = Sb, P, As) [16,21,22]. The electronic, mechanical, and structural characteristics are fundamental to the material behavior of HH alloys. First-principles calculations were employed to systematically investigate the electronic, structural, and mechanical properties of MnNiSb and MnCoZ (Z = Sb, P, As). These four compounds were selected based on their predicted ferromagnetism, distinctive optical properties, and superior thermoelectric performance, such as half-metallicity and intrinsically low lattice thermal conductivity [17]. Fracture toughness was evaluated using two different computational methods: an explicit crack model (Method I) and an empirical formula based on bulk and shear moduli (Method II) [23,24,25]. The comparison reveals different absolute values. The findings of this research are expected to further clarify the versatile performance characteristics of MnNiSb and MnCoZ (Z = Sb, P, As) and facilitate their potential engineering applications in aerospace and spintronic technologies. Moreover, this work lays a theoretical foundation for subsequent studies integrating experimental synthesis and multi-scale simulations, thereby accelerating the practical application of these materials in fields including aerospace and spintronics.

## 2. Methods and Calculation Details

All first-principles calculations in this study were performed using the VASP code, with the exchange-correlation functional treated by the PBE generalized gradient approximation (GGA) [26]. The plane-wave cutoff energy was set to 500 eV, and a 12 × 12 × 12 k-point mesh was employed for Brillouin zone integration [27]. Convergence tests performed with respect to cutoff energy and k-point mesh (using MnCoSb) showed that increasing the cutoff to 600 eV or the k-mesh to 16 × 16 × 16 changes the total energy by less than 0.5 meV/atom, confirming that the chosen parameters are well converged (see Supplementary Tables S1 and S2). The convergence threshold for electronic self-consistency was set to 10^−6^ eV, and the force tolerance for ionic relaxation was set to 0.0001 eV/Å. The projector augmented wave (PAW) method was adopted to describe the electron–ion interactions within the system [28]. Geometric structure optimization, including key structural parameters such as lattice constants and atomic positions, was carried out using the BFGS algorithm [29].

Phonon spectra were calculated using density functional perturbation theory (DFPT) as implemented in VASP. A 2 × 2 × 2 supercell containing 96 atoms was constructed, and atomic displacements of 0.015 Å were applied to compute the force constants. A 6 × 6 × 6 q-point mesh was used for integration over the Brillouin zone. The phonon dispersion curves were then interpolated along the high-symmetry path Γ–X–M–Γ–R–X using the Phonopy code.

Elastic constants were determined using the stress–strain method. Six independent strain patterns were applied with a maximum amplitude of ±0.5% in steps of 0.1%. Cubic symmetry constraints were enforced, allowing only the three independent elastic constants *C*_11_, *C*_12_, and *C*_44_ to be extracted from the linear regime of the stress–strain curves.

## 3. Results and Discussions

### 3.1. Crystal Structure and Stability

**Figure 1:**
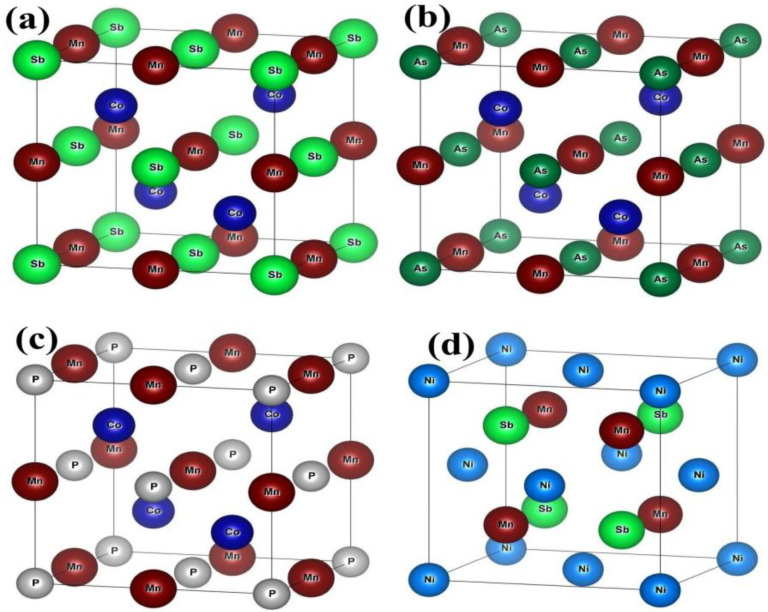
Crystal structures of the Half-Heusler(HH) alloys: (**a**) MnCoSb, (**b**) MnCoAs, (**c**) MnCoP, and (**d**) MnNiSb.

The ferromagnetic HH alloys MnNiSb and MnCoZ (Z = Sb, P, As) were selected as the subjects of this investigation. The intrinsic phases of MnNiSb, MnCoSb, MnCoP, and MnCoAs all crystallize in the cubic space group F-43m (#216), with their unit cell containing a total of 12 atoms. The alloy structure follows the XYZ-type configuration. Three distinct elements (X, Y, Z) form a cubic crystal system with a uniquely ordered arrangement (Figure 1a–d). Specifically, X, Y, and Z atoms are located at the face-centered cubic (FCC) lattice sites, tetrahedral interstitial sites, and octahedral interstitial sites, respectively. This configuration ensures the structural integrity as illustrated. In the MnCoZ (Z = Sb, P, As) system, the Sb, P, or As atoms occupy the 4a Wyckoff position (0, 0, 0), Co occupies the 4b position (1/4, 1/4, 1/4), and Mn occupies the 4c position (1/2, 1/2, 1/2) [30]. The MnCoP and MnCoA compounds were modeled by substituting the Sb atom in MnCoSb, whereas in MnNiSb, Ni occupies the 4a site (0, 0, 0), Mn the 4b site (1/4, 1/4, 1/4), and Sb the 4c site (1/2, 1/2, 1/2). The HH compounds investigated in this study feature a characteristic atomic arrangement: each elemental component forms an independent FCC Bravais lattice, creating a structure analogous to NaCl. As detailed in Table 1, the lattice parameters derived from geometric optimization show close consistency with available reference data [16,21,22,31,32], thereby validating our computational approach. This distinctive crystal structure underpins the excellent physicochemical properties observed, indicating significant potential for applications in thermoelectric and magnetic fields.

**Figure 2:**
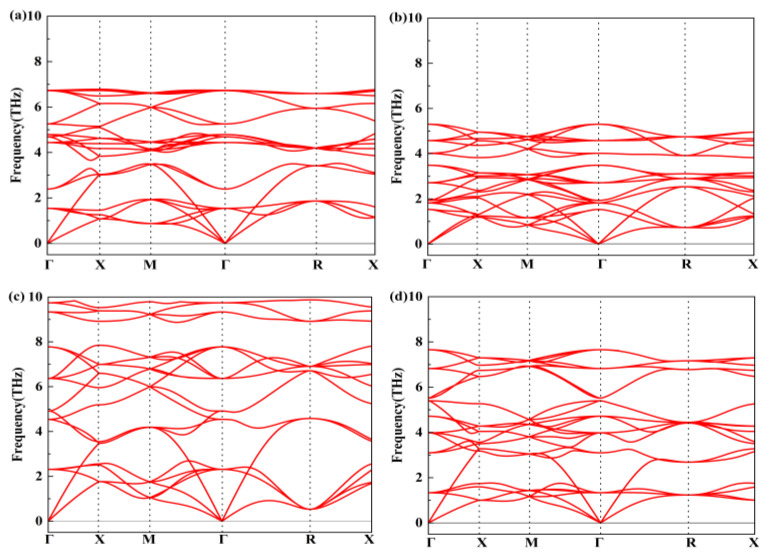
Phonon spectra of the HH alloys: (**a**) MnCoSb, (**b**) MnCoAs, (**c**) MnCoP, (**d**) MnNiSb.

Before the investigation of material properties, it is imperative to conduct a comprehensive evaluation of their stability. In this study, the phonon spectra of the four intrinsic alloys—MnNiSb, MnCoSb, MnCoP, and MnCoAswere analyzed along the high-symmetry path Γ → X → M → Γ → R → X. As shown in Figure 2a–d, the lack of imaginary component frequencies from the phonon dispersion relations confirms the dynamic stability of all four compounds, indicating their feasibility for experimental synthesis. This is consistent with the articles we cited [17,32,33,34,35,36]. Based on these results, subsequent investigations will focus on these four dynamically stable alloys.

Furthermore, the formation enthalpy was introduced as a key indicator to comprehensively evaluate the thermodynamic stability. A more negative formation enthalpy corresponds to higher stability of the compound. This trend, quantified by Equation (1) [37,38,39,40], indicates that structures with lower (more negative) formation enthalpies release more energy during formation and are more inclined to maintain their low-energy state, thereby resisting phase transitions.

(1) $$E_{\mathrm{Formation}}=E_{tot}-\sum_{i}nE_{i}$$

**Figure 3:**
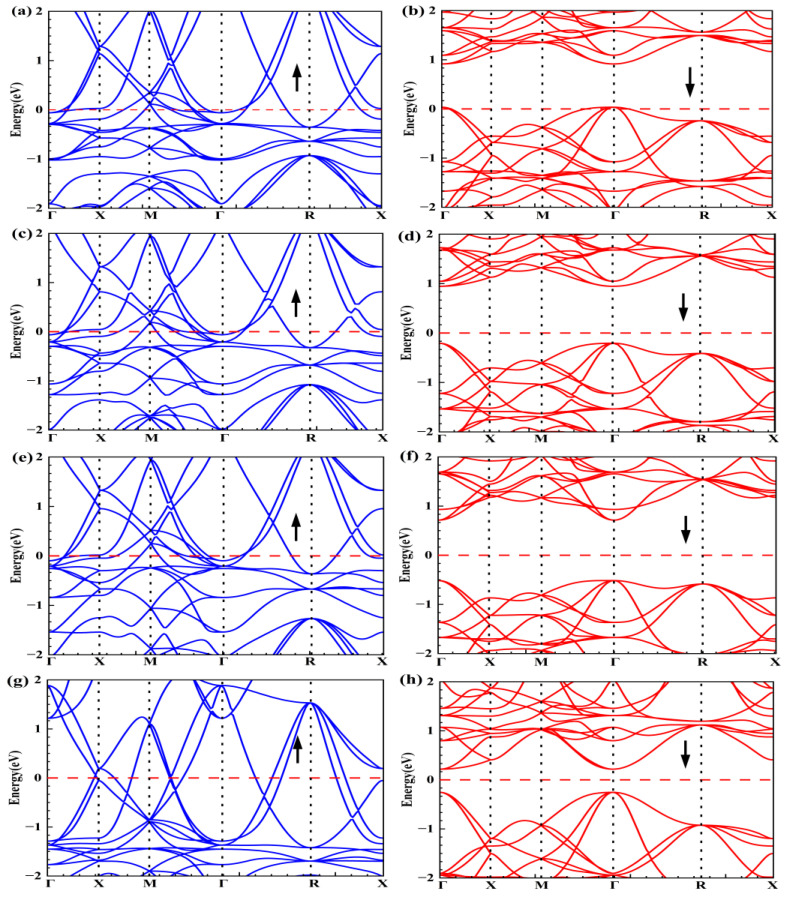
Band structures of the HH alloys: (**a**) MnCoSb (↑), (**b**) MnCoSb (↓), (**c**) MnCoAs (↑), (**d**) MnCoAs (↓), (**e**) MnCoP (↑), (**f**) MnCoP (↓), (**g**) MnNiSb (↑), (**h**) MnNiSb (↓).

Here, *E*_tot_ represents the total energy of the compound system, while *E*_i_ and *n*_i_ denote the energy of an isolated atom of the i-th element and its stoichiometric content in the unit cell, respectively. Table 1 lists all the calculated enthalpies of formation, which are consistently negative, confirming the excellent thermodynamic stability of these materials. Further analysis of the enthalpy data indicates that the calculated formation energies (Table 1) are negative for MnCoSb, MnCoAs, MnCoP, and MnNiSb, indicating that they are synthesizable and exhibit good environmental stability. Based on this characteristic, we conducted an in-depth investigation of the electronic band characteristics of the studied systems using first-principles calculations. As shown in Figure 3a–h, MnCoSb, MnCoAs, MnCoP, and MnNiSb exhibit half-metallic properties, which is consistent with previous reports on such materials [16,22,32]. The spin-up channel displays metallic characteristics, with its valence and conduction bands intersecting at the Fermi level, indicating the presence of a high concentration of mobile charge carriers. In contrast, the spin-down channel exhibits a distinct band gap at the Fermi level, suggesting semiconductor or insulator behavior for the spin-down states. This asymmetry between the spin-up and spin-down channels is a hallmark of half-metallicity and confirms the presence of ferromagnetism in all four alloys. As illustrated in Figure 3a–h, the HH alloys MnCoSb, MnCoAs, MnCoP, and MnNiSb are predicted to exhibit 100% spin polarization at the Fermi level. Consequently, these thermodynamically stable half-metallic alloys are potential candidates for spintronic devices such as spin valves, magnetic random-access memories, and spin injectors.

**Table 1:** Key computational findings, including lattice parameters, formation energy, and assessments of excellent dynamic stability under various loading conditions.

Crystal Structure Types	a = b = c (Å)	*E*_Formation_ (eV/atom)	Dynamical Stability
MnCoSb	5.812	−3.54	YES
	5.825 [34]	--	--
	5.807 [31]	--	--
	5.872 [22]	--	--
	5.835 [16]		
MnCoAs	5.532	−5.987	YES
	5.55 [22]	--	--
MnCoP	5.351	−25.556	YES
	5.37 [22]	--	--
MnNiSb	5.906	−4.324	YES
	5.93 [32]	--	--
	5.91 [21]	--	--

### 3.2. Mechanical Properties

Having confirmed the structural stability of these alloys, we further evaluated their macroscopic mechanical properties, which are essential for their potential applications as structural materials. Elastic constants [32], defined as manifestations of structural integrity, govern a material’s resistance to deformation, dictate its stress response, and establish fundamental criteria for judging the mechanical stability of the lattice [41]. Among the core mechanical characteristics, Young’s modulus (*E*), the shear modulus, and Poisson’s ratio (*v*) are crucial for assessing mechanical performance. Young’s modulus (*E*) quantitatively describes a material’s resistance to tensile and compressive deformation, with its magnitude directly reflecting the inherent stiffness of the material. The shear modulus is another critical elastic parameter; it specifically characterizes the material’s intrinsic resistance to shear deformation, thereby quantifying its dimensional stability under shear stress. Poisson’s ratio, a parameter central to elasticity theory, defines the extent of transverse deformation relative to axial deformation under tensile or compressive loading. Collectively, these elastic constants form a constitutive framework that comprehensively characterizes the fundamental mechanical behavior of a material.

The predictive power of the Born stability criteria [42,43] lies in their ability to evaluate mechanical stability across crystal systems, thereby guiding the synthesis of novel materials with targeted properties; for cubic XYZ-type HH alloys, this translates to a set of specific conditions that must be satisfied [43]:

(2) $$C_{11}>0\;,\;C_{44}>0\;,\;C_{11}+2C_{12}>0\;,C_{11}-C_{12}>0$$

The application of the data presented in Table 2 to the stability criteria confirms compliance with all necessary conditions. The calculated elastic stiffness constants for the material were determined to be the MnNiSb and MnCoZ (Z = Sb, P, As) alloys, which fulfill the theoretical requirements of the Born rule, demonstrating the mechanical robustness of the intrinsic structures of all four materials. Within the framework of the Voigt–Reuss–Hill approximation scheme [44,45,46], the bulk modulus and shear modulus [46] are computed from the derived data using the following relationships:

(3) $$B_{V}=B_{R}=\frac{C_{11}+2C_{12}}{3}\;$$

(4) $$G_{V}=\frac{C_{11}-C_{12}+3C_{44}}{5}$$

(5) $$G_{R}=\frac{5C_{44}(C_{11}-C_{12})}{4C_{44}+3\left(C_{11}-C_{12}\right)}\;$$

Among the physical quantities, such as *B*_V_, *B*_R_, *G*_V,_ and *G*_R_, the subscripts “V” and “R” represent the Voigt and Reuss approximations, respectively, the former establishing the theoretical upper bound and the latter the lower bound for the effective elastic moduli of polycrystalline materials of the Voigt–Reuss–Hill approximation method. By taking the arithmetic average of the values derived from the Voigt and Reuss models, one obtains the Hill model [38]:

(6) $$B_{H}=\frac{B_{V}+B_{R}}{2}\;$$

(7) $$G_{H}=\frac{G_{V}+G_{R}}{2}\;$$

The values of *E* and *ν* are determined through the rigorous application of foundational theoretical relations connecting them to the parameters *B* and *G* [47]:

(8) $$E=\frac{9GB}{G+3B}\;$$

(9) $$v=\frac{3B-2G}{6B+2G}\;$$

The Vickers hardness [48,49] is defined as a fundamental metric for evaluating a material’s resistance to deformation, quantified by the following formula [48]:

(10) $$H_{v}=0.92*k^{1.137}*G^{0.708}$$

Here, k=G/B.

The Cauchy stress-defined pressure (*C*_P_), a variable widely used in the intersection of materials science and solid mechanics, functions as a key parameter for predicting the ductile versus brittle behavior of a material. For intermetallic compounds and complex alloys, the Cauchy index serves as an indicator for the ductile versus brittle behavior and provides significant practical utility by offering a metric to predict the mechanical response, particularly the ductile versus brittle character: $C_{P}=C_{12}-C_{44}$ [50]. Substituting the *C*_12_ and *C*_44_ values of the alloy’s materials from Table 2 into this formula yields positive Cauchy pressure values. The observed enhancement in ductility implies that deformation is primarily accommodated by dislocation glide, suppressing brittle fracture.

The calculated mechanical parameters, including the bulk modulus (*B*), shear modulus (*G*), Young’s modulus (*E*), Poisson’s ratio (*ν*), Cauchy pressure, *B*/*G* ratio, Cauchy pressure (*C*_p_), and Vickers hardness (*H*_v_), are systematically summarized for the alloy materials in Table 3.

**Figure 4:**
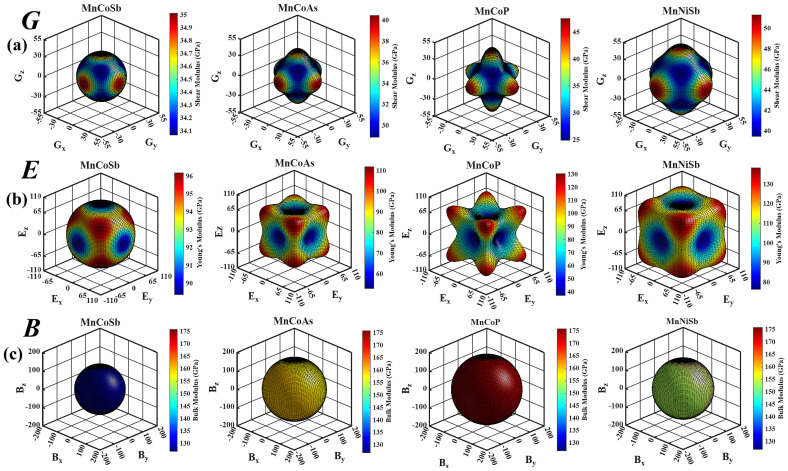
Three-dimensional elastic moduli of the HH alloys: (**a**) shear modulus (*G*), (**b**) Young’s modulus (*E*), (**c**) bulk modulus (*B*).

Based on (DFT) calculations, the intrinsic elastic moduli and related physical properties of MnNiSb and MnCoZ (Z = Sb, P, As) Heusler alloys were obtained, with the calculated results summarized in Table 3. As two key macroscopic mechanical parameters, the bulk modulus (*B*) and shear modulus (*G*) are directly determined from the set of fundamental elastic constants. They serve as core parameters for evaluating a material’s resistance to volumetric and shear deformation, where the bulk modulus represents the resistance to volume change under elastic deformation, and the shear modulus reflects the resistance to shape change under plastic deformation. Among the alloys investigated, MnCoP exhibits the highest bulk modulus (175.87 GPa), indicating its excellent compressive resistance and high stiffness. In contrast, MnCoSb shows the lowest bulk modulus (126.91 GPa). Regarding the shear modulus (*G*), which reflects the material’s resistance to plastic deformation, MnNiSb demonstrates a value (39.64 GPa) considerably higher than those of the other three compounds, suggesting that dislocation motion in MnNiSb may be more difficult, potentially affecting its plastic deformation mechanism. Additionally, Young’s modulus (*E*), an important parameter for assessing tensile stiffness, varies significantly among these alloys, consistent with the overall trends observed for the bulk modulus (*B*) and shear modulus (*G*). As illustrated in Figure 4, three-dimensional surface representations of the shear (Figure 4a), bulk (Figure 4b), and Young’s (Figure 4c) modulus are presented. MnCoSb exhibits a moderate bulk modulus (126.91 GPa) but high mechanical anisotropy, as reflected by its three-dimensional modulus surfaces, whereas MnCoAs and MnNiSb exhibit progressively less mechanical anisotropy, and MnCoP shows the most pronounced mechanical anisotropy, consistent with the data summarized in Table 3. The combination of a high bulk modulus and a high Young’s modulus implies that these materials can maintain structural integrity when employed as structural components or subjected to stress conditions. Furthermore, a comprehensive understanding of the anisotropy of Young’s modulus is crucial for the future design of microelectronic or functional devices based on these materials, as device performance may be influenced by grain orientation and the direction of applied stress fields. This favorable mechanical stability, together with the thermodynamic stability and half-metallic electronic characteristics discussed above, collectively establishes the potential of these HH alloys for applications in advanced spintronic devices.

The ductile or brittle behavior of materials can be empirically assessed using Poisson’s ratio (*v*) and the *B*/*G* ratio. According to Pugh’s criterion, a *B*/*G* ratio greater than 1.75 typically indicates ductile behavior, whereas a lower value suggests brittleness. In this study, the *B*/*G* ratios of all four alloys significantly exceed 1.75, ranging from 1.93 to 2.50, and their Poisson’s ratios (*v*) are all greater than 0.3. These results indicate that MnNiSb, MnCoSb, MnCoP, and MnCoAs are expected to exhibit good ductility. This finding is supported by the fact that their Cauchy pressures (*C*_p_) are all significantly positive, further confirming their ductile behavior and the dominance of metallic bonding in these alloys. However, it is worth noting that although MnNiSb has a *B*/*G* ratio of 1.97 and a Poisson’s ratio of 0.38, its relatively high shear modulus suggests that it may exhibit a stronger tendency toward brittleness compared to the other three alloys, possibly implying mixed metallic–covalent bonding characteristics. It should be emphasized that all four alloys remain within the ductile regime according to the Pugh criterion; the differences among them represent a spectrum within ductility rather than a transition to brittle behavior. MnNiSb is simply less ductile (i.e., relatively more brittle) than the others, but it is still ductile overall. In terms of stiffness, the calculated Young’s modulus (*E*) shows that MnNiSb possesses the highest value (109.48 GPa), indicating its strongest resistance to elastic deformation, whereas MnCoP exhibits the lowest stiffness (80.25 GPa). Regarding Vickers hardness (*H*_v_), the values for the four alloys fall within a narrow range (23.61–27.7 GPa). Overall, the theoretical calculations suggest that MnCoSb, MnNiSb, MnCoP, and MnCoAs are all predicted to exhibit good ductility.

### 3.3. Electronic Structure

**Figure 5:**
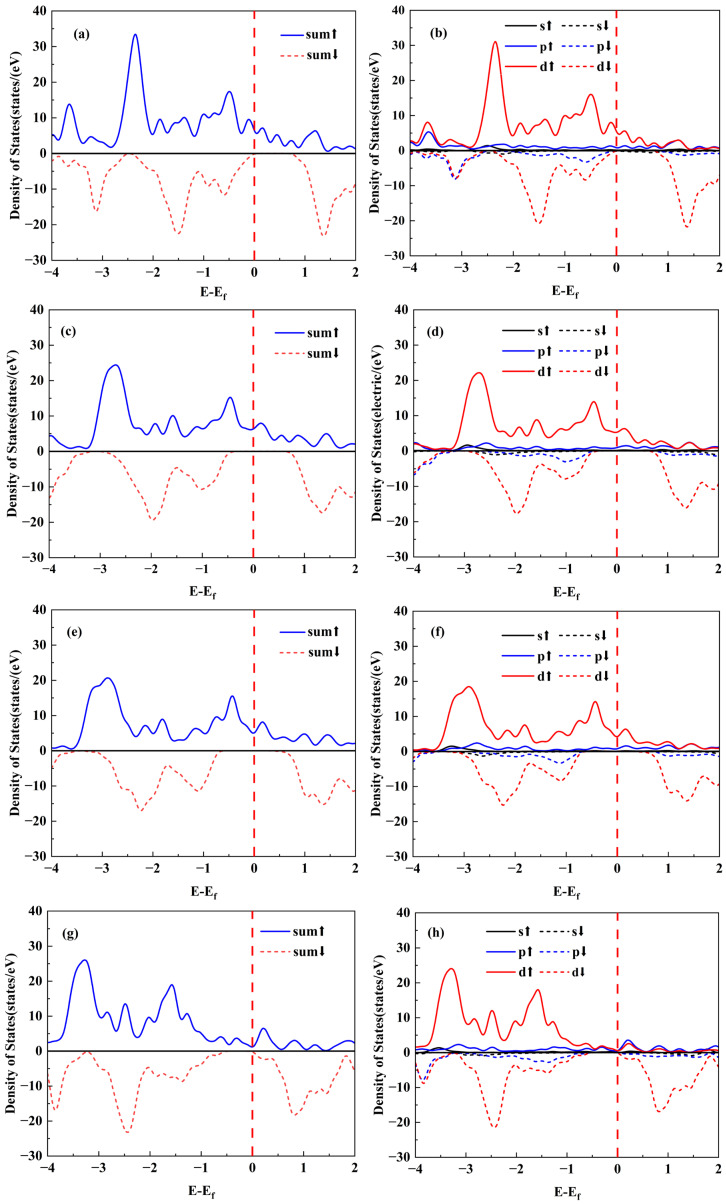
Density of states of the (**a**) MnCoSb Total, (**b**) MnCoSb DOS, (**c**) MnCoAs Total, (**d**) MnCoAs PDOS, (**e**) MnCoP Total, (**f**) MnCoP PDOS, (**g**) MnNiSb Total, and (**h**) MnNiSb PDOS. HH alloys.

**Figure 6:**
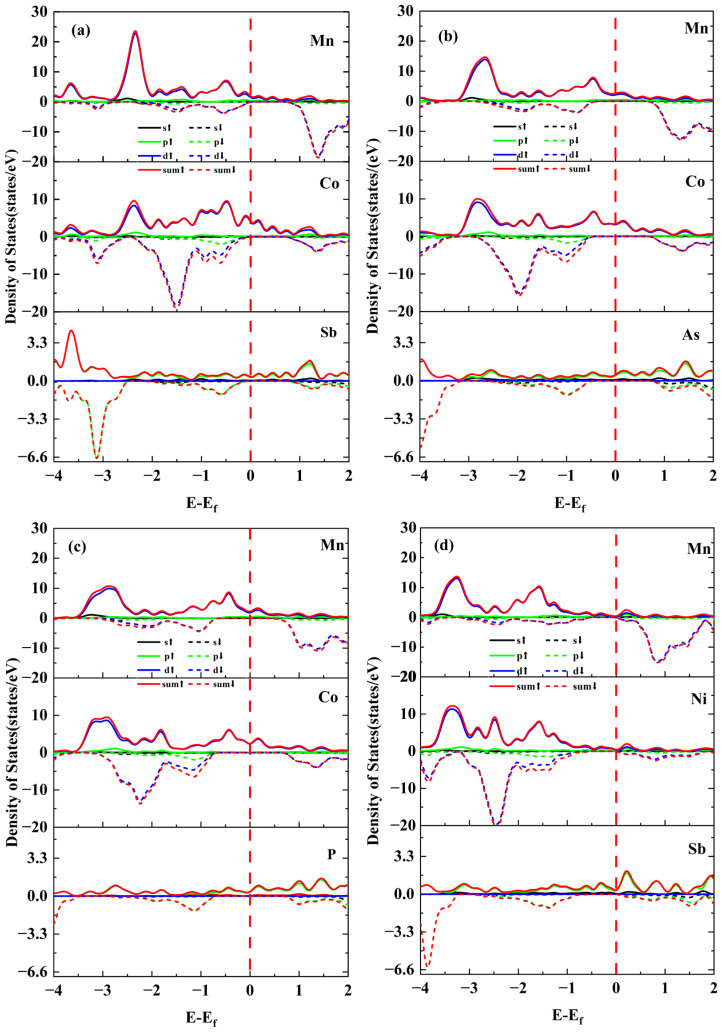
Element-projected density of states (PDOS) of the (**a**) MnCoSb, (**b**) MnCoAs, (**c**) MnCoP, and (**d**) MnNiSb. HH alloys.

The differences in macroscopic mechanical properties essentially originate from the microscopic electronic structure and chemical bonding characteristics of the materials. To further understand the aforementioned mechanical behavior, particularly the differences in the ductility–brittleness tendency between MnNiSb and the other alloys, we calculated and analyzed the density of states (DOS) of all investigated systems. The variations in mechanical properties are primarily attributed to the influence of different elemental compositions on the electronic structure and interatomic bonding characteristics [51,52,53]. As confirmed by the electronic DOS results of the alloys shown in Figure 5, the fundamental reason for the differences in macroscopic mechanical properties lies in the unique electronic structures and chemical bonding characteristics arising from their distinct chemical compositions. The DOS analysis reveals that all systems exhibit residual DOS at the Fermi level, indicating their metallic nature. Specifically, for MnCoSb (Figure 5a,b), the DOS distribution near the Fermi level is relatively steep, with the Mn-*d* and Sb-*p* orbitals (Figure 6a) showing strong hybridization peaks and a high degree of energy overlap. This suggests the presence of strong directional covalent bonds between atoms, which is closely related to the alloy’s high elastic modulus and inherent brittleness. In contrast, MnNiSb, MnCoP, and MnCoAs exhibit a flatter DOS distribution near the Fermi level, indicating greater electron delocalization [54,55] and thus more pronounced metallic bonding characteristics.

A correlation analysis was subsequently conducted between the electronic configurations and macroscopic mechanical properties of the HH alloys (MnNiSb, MnCoAs, MnCoSb, MnCoP). The element-resolved DOS results shown in Figure 6a–d demonstrate that different elemental compositions have a decisive influence on the distribution of electronic states near the Fermi level. The Mn-*d* orbitals (Figure 6a–d) exhibit significant spin polarization in the energy range from −2 to 1 eV, with the spin-up and spin-down d orbitals distinctly separated, reflecting the crystal field splitting effect, and thus the electronic origin of the magnetic anisotropy in these materials [56,57]. Notably, the difference between the transition metals Co (Figure 6a–c) and Ni (Figure 6d) plays a significant role in modulating the electronic structure: the Co-3*d* orbitals give rise to new electronic states near the Fermi level, shifting and broadening the DOS peaks. The *d*-*d* coupling likely enhances the metallic character of the material, which is consistent with theoretical trends in electrical conductivity [58].

Furthermore, the differences in spin-resolved DOS at the Fermi level are particularly pronounced for the P-based system (Figure 6c), suggesting that certain compositions may exhibit half-metallicity, where electrons of one spin orientation display metallic behavior while those of the opposite spin exhibit insulating characteristics. This property holds great potential for spintronic applications and further confirms that MnCoP and MnCoAs, obtained by substituting Sb in MnCoSb, retain the half-metallic nature of the parent compound. The difference in the ductility–brittleness tendency of the MnNiSb alloy is directly reflected in its DOS plots (Figure 5g,h): a prominent peak originating from the Ni-3d orbitals is observed at 1–2 eV above the Fermi level. This corresponds to the occupation of antibonding states arising from the additional d electrons of Ni compared to Co [59]. The occupation of these antibonding states directly weakens the net bond strength of the Mn-Ni and Ni-Sb bonds, leading to changes in elastic moduli, higher stiffness, and a tendency toward brittleness in terms of the ductility brittleness tendency [54,55]. MnCoP exhibits the most distinctive mechanical behavior, with the lowest strength and most pronounced anisotropy, but potentially superior plastic deformability. Its DOS plots show a moderate total DOS at the Fermi level, and the *d*-band centers of Mn and Co are located farther from the Fermi level compared to the other systems. Meanwhile, the hybridization peaks between the P *p*-orbitals, Figure 6c, and the transition metal *d*-orbitals are broader and shallower. This electronic structure suggests a bonding nature with less covalency and a stronger tendency toward metallicity. The weaker bond directionality likely allows dislocations to nucleate under lower stresses, while the metallic character facilitates dislocation slip and multiplication. Consequently, more significant plastic flow during deformation can dissipate energy, potentially contributing to greater fracture strain [60,61].

Magnetic properties. Since all four alloys exhibit half-metallic ferromagnetism, we further calculated their total magnetic moments and atomic-resolved contributions. The total magnetic moments per formula unit are MnCoSb = 4.00 μ_B_, in agreement with the literature [16,17,22]; MnCoAs = 4.00 μ_B_ [22]; MnCoP = 3.95 μ_B_ [22]; and MnNiSb = 4.00 μ_B_ [32]. The magnetic moments are mainly contributed by the Mn atoms (≈3.2–3.5 μ_B_), while Co and Ni atoms provide small opposite contributions (≈−0.2 μ_B_); the *p*-block elements exhibit negligible magnetic moments. These values follow the Slater–Pauling rule (*Z*_t_ − 18 = 4 μ_B_) [32]. To assess the influence of magnetism on elastic constants, we performed a control calculation for MnCoSb by switching off spin polarization (non-magnetic state). The resulting elastic constants *C*_11_ = 170.32 GPa, *C*_12_ = 105.88 GPa, and *C*_44_ = 35.40 GPa change by less than 5%, and the *B*/*G* ratio remains above 1.75. Hence, magnetism does not dominate the mechanical behavior; the ductile nature and fracture toughness reported herein are robust against magnetic ordering. A similar weak dependence of elastic constants on magnetism has been observed in other HH systems [32,62].

In summary, the range of macroscopic mechanical properties (strength, plasticity) of these HH alloys is precisely governed by the DOS distribution near the Fermi surface and the intensity and characteristics of *p*-*d* orbital hybridization. Strong hybridization and a high DOS at the Fermi level promote high strength and high stiffness, potentially at the expense of ductility, whereas moderate hybridization combined with enhanced metallic character favors a better strength–ductility synergy. The electronic origin of these property differences ultimately determines the material’s resistance to crack propagation, i.e., its fracture toughness.

### 3.4. Fracture Toughness

The fracture resistance of a material is crucial to its service safety and structural reliability. For load-bearing alloys, their ability to resist unstable crack propagation is commonly characterized by the plane-strain fracture toughness (*K*_IC_), i.e., the critical stress intensity factor. The theoretical basis of this parameter stems from Griffith’s energy balance theory [63], which states that the significant discrepancy between the practical and theoretical fracture strengths of materials originates from stress concentration at the tips of pre-existing microcracks under external loading. Crack propagation becomes unstable when the elastic strain energy released by the system due to crack extension is sufficient to compensate for the energy required to create new fracture surfaces, a critical condition known as the Griffith criterion. From an engineering perspective, fracture toughness *K*_IC_ is an important mechanical property that quantifies a material’s resistance to rapid fracture containing defects or cracks. It is defined as the critical stress intensity factor, *K*_IC_. A higher *K*_IC_ value indicates a greater ability to suppress crack growth, thereby enhancing component safety [64,65].

Currently, the calculation and assessment of material fracture toughness mainly rely on two mainstream methods. The first one is a simple method, which only requires obtaining the bulk modulus and shear modulus of the material to conduct the calculation; the second one is a refined method, which requires first constructing a two-dimensional crack model of the material and then completing the assessment through quantitative calculation of the crack formation energy. The explicit crack model (Method I) is constructed as follows [40,66,67]:

(11) $$K_{\mathrm{IC}}=\sqrt{\frac{G_{IC}*E}{1-v^{2}}}\;$$

In this study, the specific crystallographic direction (001) was selected as a potential cleavage plane, and a crack model was constructed accordingly. Specifically, a 1 × 1 × 1 supercell was used, with a vacuum layer of 15 Å added perpendicular to the crack plane to avoid periodic interactions. All atoms within a 5 Å radius of the crack tip were fully relaxed until the residual forces were less than 0.01 eV/Å. The crack formation energy *E*_crack_ was then calculated as the energy difference between the cracked and pristine supercells, divided by the crack area *A*_crack_.

(12) $$G_{IC}=\frac{E_{crack}-E_{initial}}{A_{crack}}\;$$

where *E*_crack_ represents the crack formation energy in electron volts (eV), defined as the energy difference between the cracked and initial systems; *E*_initial_ denotes the total energy of the pristine crystal structure in eV; and *A*_crack_ indicates the cross-sectional area of the crack in square angstroms (Å^2^).

The empirical formula (Method II) is as follows [68]:

(13) $$K_{\mathrm{IC}}=V_{0}^{1/6}G{\left(B/G\right)}^{1/2}$$

Here, *V*_0_ is the volume of the primitive cell (*V*_0_ = a × b × c), and *B* and *G* are the bulk and shear moduli.

To evaluate damage tolerance, the brittle index *M* is calculated as [39,68]

(14) $$M=H_{V}/K_{\mathrm{IC}}$$

The damage tolerance of a material can be assessed by the brittle index *M*, a parameter that includes the Vickers hardness, with a lower *M* value denoting superior tolerance. As summarized in Table 4, the key parameters required for calculating the mode I fracture toughness (*K*_IC_) via two different methods are presented, along with the final results. The two methods yield different absolute values (e.g., a difference of about 40% for MnCoSb). Therefore, the explicit crack model (Method I) is preferred for quantitative assessment of fracture toughness in anisotropic HH alloys, whereas the empirical formula (Method II) should be used only as a rough comparative reference or for preliminary screening.

In the study of HH alloys, accurate evaluation of fracture toughness (*K*_IC_) and damage tolerance is crucial for assessing their potential as structural–functional integrated materials. This study presents two theoretical methods for estimating *K*_IC_ based on intrinsic material parameters (atomic volume *V*_0_, bulk modulus *B*, and shear modulus *G*), establishing a bridge between electronic/atomic-scale calculations and macroscopic fracture properties. To further quantify the brittle ductile characteristics, the brittleness index *M* is introduced, where lower values indicate superior damage tolerance and enhanced resistance to brittle fracture while maintaining high hardness.

As summarized in Table 4 for the MnNiSb and MnCoZ (Z = Sb, P, As) alloy systems, the calculated results indicate that among these four alloys, both models confirm that MnCoSb exhibits the most outstanding comprehensive performance. It possesses the lowest brittleness index (*M* = 8.97 ^I^, 14.94 ^II^) and relatively high fracture toughness (*K*_IC_ = 2.63 ^I^ MPa·m^1/2^, 1.58 ^II^ MPa·m^1/2^), suggesting excellent damage tolerance. In contrast, MnCoP exhibits the lowest *K*_IC_ values (2.00 ^I^ MPa·m^1/2^,1.63 ^II^ MPa·m^1/2^) and the highest *M* values among the four half-metals (13.85 ^I^, 16.99 ^II^), implying a greater tendency toward brittle fracture and potentially inferior mechanical reliability. The properties of MnNiSb (*K*_IC_ = 2.55 ^I^ MPa·m^1/2^,1.90 ^II^ MPa·m^1/2^; *M* = 10.54 ^I^, 14.14 ^II^) and MnCoAs (*K*_IC_ = 2.28 ^I^ MPa·m^1/2^, 1.62 ^II^ MPa·m^1/2^; *M* = 11.61 ^I^, 16.32 ^II^) lie between these two alloys. Overall, the fracture toughness values of the four half-metallic materials are consistent with the expected calculated results. The comparison between the two methods, despite differences in absolute values, confirms that the explicit crack model (Method I) is more reliable for anisotropic systems because it directly simulates crack propagation, accounts for local atomic relaxations, and does not rely on isotropic assumptions. In contrast, Method II serves as a rough comparative reference due to its simplicity. The ability to quantitatively predict key mechanical properties of XYZ-type intermetallic compounds through DFT calculations provides critical parameters and theoretical insights. This enables the rational design and strategic screening of novel alloys with excellent damage tolerance based on fundamental theoretical principles.

## 4. Conclusions

A systematic evaluation of the HH alloys MnCoSb, MnCoAs, MnCoP, and MnNiSb using DFT confirms their excellent thermodynamic and mechanical stability. The negative formation energies and the absence of imaginary frequencies in the phonon spectra verify their synthesizability and dynamic stability under ambient conditions. Furthermore, their elastic constants satisfy the Born stability criteria for cubic crystals, thereby ensuring mechanical stability. The fracture toughness of all alloys lies in the range of 1.58 MPa·m^1/2^–2.63 MPa·m^1/2^, and their Poisson’s ratios and *B*/*G* ratios exceed the critical value of 1.75. Notably, MnNiSb exhibits a relatively high tendency toward brittleness. Nevertheless, all alloys satisfy the B/G > 1.75 criterion, confirming that they are all ductile; the observed differences reflect a spectrum within the ductile regime. The higher tendency toward brittleness of MnNiSb is attributed to its relatively high shear modulus (39.64 GPa) and the significant covalent bonding character revealed by its electronic structure. Electronic structure characterization confirms the half-metallic nature of all compounds, with a theoretical spin polarization of 100%. For fracture toughness, the explicit crack model (Method I) is physically more realistic for anisotropic HH alloys, while the empirical formula (Method II) serves as a convenient reference. Although the two methods give different absolute values, the comparison confirms that Method I is preferred for quantitative assessment. The results indicate that MnCoSb exhibits the most outstanding overall performance, demonstrating the lowest brittleness index *M* (8.97, 14.94) and relatively high *K*_IC_ values (2.63 MPa·m^1/2^, 1.58 MPa·m^1/2^), suggesting its excellent damage tolerance. Among the other three half-metals, MnCoAs shows *K*_IC_ values of 2.28 MPa·m^1/2^ and 1.62 MPa·m^1/2^ and *M* values of 11.61 and 16.32; MnCoP exhibits *K*_IC_ values of 2.00 MPa·m^1/2^ and 1.63 MPa·m^1/2^ and the highest *M* values of 13.85 and 16.99; and MnNiSb shows *K*_IC_ values of 2.55 MPa·m^1/2^ and 1.90 MPa·m^1/2^ and *M* values of 10.54 and 14.14. The fracture toughness results for the four half-metallic materials indicate that MnCoP exhibits a greater tendency toward brittleness compared to the other three half-metals, while MnCoSb demonstrates the most favorable overall properties. This study achieves a quantitative prediction of the fracture toughness of HH alloys through first-principles calculations, revealing the excellent fracture toughness and damage-tolerant nature of the MnCoSb HH alloy, thereby establishing a theoretical foundation for the design of functional materials with excellent damage tolerance. The proposed methodology and model provide a paradigm for the rational design of high-performance materials integrating structural and functional properties.

## Figures and Tables

**Table 2:** The calculated independent elastic stiffness coefficients (*C*_ij_), MnNiSb, MnCoZ (Z = Sb, P, As).

Crystal Structure Types	*C*_11_	*C*_12_	*C*_44_
MnCoSb	170.20 [35]	105.36	35.02
	175.08 [31]	72.20 [31]	35.80 [31]
	191.56 [35]	76.66 [35]	57.57 [35]
MnCoAs	184.81	148.10	40.46
MnCoP	192.90	167.36	47.52
MnNiSb	189.29	135.32	51.29
	167.07 [36]	82.06 [36]	53.27 [36]
	138 [32]	45 [32]	48 [32]

**Table 3:** Based on the computational results, the key mechanical parameters for the alloys MnNiSb and MnCoZ (Z = Sb, P, As) are summarized below. These parameters include the bulk modulus (*B*), Cauchy pressure (*C*_p_), Young’s modulus (*E*), shear modulus (*G*), Poisson’s ratio (*ν*), *B*/*G* ratio, and Vickers hardness (*H*_v_).

Crystal Structure Types	*B* (GPa)	*G* (GPa)	*E* (GPa)	*v*	*C*_p_ (GPa)	*B*/*G*	*H*_v_ (Pa)
MnCoSb	126.91	33.91	98.97	0.38	70.35	1.93	23.61
	145.0 [31]	43.0 [31]	114.56 [31]	0.33 [31]	--	--	--
MnCoAs	160.34	29.46	83.28	0.41	107.64	2.33	26.44
MnCoP	175.87	28.18	80.25	0.42	119.86	2.50	27.70
MnNiSb	153.31	39.64	109.48	0.38	84.03	1.97	26.87
	110.4 [36]	48.67 [36]	127.3 [36]	0.308 [36]	--	2.268 [36]	--

**Table 4:** The computed crack area *A*_crack_, 2D shear modulus *G*^2D^, unit cell volume *V*_0_, Young’s modulus *E*^2D^, critical energy release rate *G*_IC_, *K*_IC_, and brittle index *M*.

Compounds	*A*_crack_ (Å^2^)	*V*_0_ (Å^3^/atom)	*G*^2D^ (GPa)	*E*^2D^ (GPa)	*G*_IC_ (J/m^2^)	*K*_IC_ (Mpa·m^1/2^)		*M*	
MnCoSb	5.81079	196.13	64.58	89.39	66.18	2.63 ^I^ [40]	1.58 ^II^	8.97 ^I^	14.94 ^II^
MnCoAs	5.327102	151.17	60.17	53.05	78.48	2.28 ^I^	1.62 ^II^	11.61 ^I^	16.32 ^II^
MnCoP	5.351484	153.30	58.77	37.45	83.87	2.00 ^I^	1.63 ^II^	13.85 ^I^	16.99 ^II^
MnNiSb	5.90584	206.01	77.56	76.47	70.26	2.55 ^I^	1.90 ^II^	10.54 ^I^	14.14 ^II^

^I.^ Method I (explicit crack model) using *G*_IC_, *E*, and *ν*; ^II.^ Method II (empirical formula) using *B* and *G*.

## Data Availability

This manuscript has associated data in a data repository. The raw data supporting the conclusions of this article will be made available by the authors on request.

## Supplementary Materials

The following supporting information can be downloaded at https://www.mdpi.com/article/10.3390/molecules31121994/s1, Table S1: Convergence of total energy (in eV) with respect to plane wave cutoff energy for the four HH alloys, with the k point mesh fixed at 12 × 12 × 12. The optimized lattice constants a = b = c (Å) are also listed; Table S2: Convergence of total energy (in eV) with respect to k point mesh for the four HH alloys, with the plane wave cutoff energy fixed at 500 eV. The optimized lattice constants a = b = c (Å) are also listed.
