# Supplementary material for: Unveiling Superior Fracture Toughness in MnCoSb Half-Heusler Alloy: A First-Principles Guide for Designing Damage-Tolerant Functional Materials
Source: Molecules. 2026 Jun 7;31(12):1994. doi: 10.3390/molecules31121994 (PMC13304688; doi:10.3390/molecules31121994)
Supplement: Supplementary file 1 [file molecules-31-01994-s001.zip › molecules-4333171-supplementary.pdf]

Table S1: Convergence of total energy (in eV) with respect to plane-wave cutoff energy for the four half-Heusler alloys, with the k-point mesh fixed at 12×12×12. The optimized lattice constants a = b = c (Å) are also listed.

Crystal structure types	450 (eV)	500 (eV)	600(eV)	a = b = c (Å)
MnCoSb	-81.8098	-81.8130	-81.8104	5.81
MnCoAs	-85.2175	-85.2012	-85.2046	5.53
MnCoP	-89.8606	-89.8539	-89.8604	5.35
MnNiSb	-76.8414	-76.8574	-76.8444	5.90

Note: The total energy difference between 500 eV and 600 eV is within 1 meV/atom for all alloys, confirming that 500 eV is sufficiently converged.

Table S2: Convergence of total energy (in eV) with respect to k-point mesh for the four Half-Heusler alloys, with the plane-wave cutoff energy fixed at 500 eV. The optimized lattice constants a = b = c (Å) are also listed.

Crystal structure types	8×8×8	12×12×12	16×16×16	a = b = c (Å)
MnCoSb	-81.8111	-81.8130	-81.8108	5.81
MnCoAs	-85.1981	-85.2032	-85.1977	5.53
MnCoP	-89.8475	-89.8539	-89.8464	5.35
MnNiSb	-76.8489	-76.8574	-76.8436	5.90

Note: The total energy difference between 12×12×12 and 16×16×16 is less than 0.5 meV/atom, confirming that 12×12×12 is well converged.
